# Vision screening as part of the school health policy in South Africa from the perspective of school health nurses

**DOI:** 10.4102/phcfm.v14i1.3172

**Published:** 2022-02-07

**Authors:** Thokozile I. Metsing, Wanda E. Jacobs, Rekha Hansraj

**Affiliations:** 1 Discipline of Optometry, Faculty of Health Sciences, University of Johannesburg, Johannesburg, South Africa; 2 Discipline of Nursing, Faculty of Health Sciences, University of Johannesburg, Johannesburg, South Africa; 3 Discipline of Optometry, Faculty of Health Sciences, University of KwaZulu-Natal, Durban, South Africa

**Keywords:** vision screening, school health policy, school performance, school-going age, visual acuity, accommodative dysfunctions, school health nurse, optometrists, academic performance, Department of Health

## Abstract

**Background:**

Vision screenings of school-going children are essential in the early detection of visual anomalies common in different age categories, which may negatively affect their academic ability and social development. Hence, their inclusion in school health policies is imperative. The aim of this study was to assess the implementation of vision screening protocols in the current Integrated School Health Screening policy of South Africa from the perspective of school health personnel.

**Aim:**

The study sought to explore the perceptions, experiences and attitudes of the school health nurses on vision screenings included as part of the school health screenings in Gauteng province (South Africa).

**Setting:**

This study was located across three public healthcare facilities across Gauteng at primary healthcare levels.

**Methods:**

Three teams of 13 school health personnel from three primary healthcare facilities in the district of Ekurhuleni were invited to participate in the study. Focus group interviews were conducted for generating information on collective opinions and the rationale behind their views.

**Results:**

Results of the collected qualitative data revealed challenges related to training, vision screening tests, referral criteria and follow-ups or referral pathways. In addition, further challenges reported were related to communication, time, space and consent forms not signed by the parents.

**Conclusion:**

Improved cohesion and communication between all role players will enable reasonable and professional provision of validated vision screening services that have the best chance of early detection of children with vision anomalies to negate possible adverse effects on their scholarly performance.

## Introduction

South Africa has poor educational outcomes and high rates of grade repetition by international standards, compared with other developing states as recorded in numerous studies.^1^ The dropout rate of learners from schools is attributed to various factors including health problems amongst others such as learning disabilities, educational systemic factors or social economic and political framework.^2^ A school health policy involving health screenings was thus implemented in 2002 and reviewed in 2012.^3,4^ The implemented policy was aimed at the improvement of the general health of school-going children, to address visual health barriers to learning and to improve education outcomes with the intention of improving pass rates and learner retention within schools. The health screening of each learner as outlined in the policy includes anthropometric screening, assessments of oral health, speech, basic hearing, fine and gross motor problems, chronic illnesses (including tuberculosis and human immunodeficiency virus and/or acquired immunodeficiency syndrome [HIV/AIDS]), psychosocial risk assessments and vision.

The South African integrated school health policy states that professional nurses should be appointed as leaders of school health teams, with the recommendation of one professional nurse for every 2000 learners. In addition, the enrolled nurses or enrolled nursing auxiliary can also be appointed to assist in conducting the school health screenings including vision screenings. However, in terms of vision screenings, different eye care professionals such as private optometrists, ophthalmologists, optical dispensers and medical practitioners also conduct them in South Africa for children of school-going age (6–19 years old). Furthermore, school vision screenings are conducted on an ad hoc basis and in an uncoordinated manner, thus there is a lack of epidemiologic data on prevalent visual anomalies amongst school-going children.^4^

In the study conducted by Clarke-Farr,^5^ the need for the training of school health nurses (SHN) in conducting the appropriate vision screening procedures was identified. In the study,^5^ the nurses interviewed indicated that the vision screening largely focused on the measurement of visual acuity (VA) and external observation of eyes. Furthermore, the study found that SHNs conducting vision screenings had not undergone any formal training in the eye and vision care, and most of the evaluation skills were acquired by ‘on the job’ training, which may often result in over/under-referrals.^5^ Evidence indicates that public healthcare nurses with adequate training delivered high positive predictive values compared with the lay volunteers and other healthcare professionals.^6^

The findings of numerous other studies conducted in South Africa also indicated that despite the implemented schools vision screenings there are still children in mainstream schools with visual anomalies such as accommodative and convergence dysfunctions that negatively impact their academic performance.^6,7,8,9,10,11,12^ The unnecessary frustrations experienced by children with these visual anomalies may lead to children dropping out of school, currently a challenge encountered by the South African education system.^1^ The aim of this study was to investigate the perceptions, experiences and attitudes of the SHNs on vision screenings included as part of the school health screenings in South Africa.

## Methods

### Study design

A qualitative research design that was exploratory, descriptive and contextual with a phenomenological approach was used in the research. Phenomenological approach was utilised that translated into gathering in-depth information and perceptions from those involved in the execution of school vision screenings.^13^ This approach was used to illuminate specifics relating to the strengths and weaknesses of the vision screenings included in the school health programme, as perceived by SHNs.

### Setting

The study was located across three Ekurhuleni primary healthcare facilities in the Gauteng province, where one of the services been provided was school health services by SHNs, school health promotors, auxiliary nurses and school health co-ordinators.

### Study population and sampling strategy

The study population consists of auxiliary nurses, SHNs, school health co-ordinators and school health promotors who participated in school health screenings. The participants were purposively selected. The inclusion criteria for the participants were experience in school health screening; experience in school vision screening and consent to participate in the research. The exclusion criteria were all those SHNs, co-ordinators and auxiliary nurses not experienced or involved in school health screening and school vision screening. Three teams of 13 school health personnel from a district in the province of Gauteng were divided into three teams consisting of the following:

- School health nurses (professional nurses) (*n* = 10)
- School health promoter (*n* = 1)
- Auxiliary nurse (*n* = 1)
- School health co-ordinator (*n* = 1)

The study participants were heterogynous with 12 females and 1 male and they were only African. The participants complying with the inclusion criteria from a district in the province of Gauteng were telephonically invited through their team leaders to participate in the study. The first and second groups of participants consisted of four members and the third group had five participants. The researcher facilitated the focus groups and concerted effort in allowing each of the participants an opportunity to voice their contribution.

### Data collection

Focus group discussions were conducted within the guidelines described by Krueger and Casey^13^ relating to sequence preparation, environmental setting and the role of the researcher. The role of the researcher in the focus group discussions was to facilitate the discussions and to use communication techniques such as probing, clarifying, rephrasing to elicit information on collective opinions and the meanings lying behind the views of the school health personnel.^13,14^ The focus groups were conducted in English as it is the language used by the school health personnel. The venues for the focus group interviews were the offices of the school health personnel regarded as private by the participants. An open-ended question was used such as ‘what are your experiences in conducting vision screening tests included in the school health policy?’ The focus group discussions were held over a period of three days. The themes discussed were on training, vision-screening tests included in the ISHP, human resources capacity and coverage, referral criteria and follow-up pathways of those found to have visual anomalies. The perceptions collected from the school health personnel assisted the researcher in clarifying, extending, qualifying or challenging the objectives of the vision screenings included in the school health policy. The focus group discussions were conducted until adequate saturation of data was reached. The researcher played the role of moderator and conducted discussions using a quality audio recorder to collect data.

### Data analysis

**FIGURE 1:**
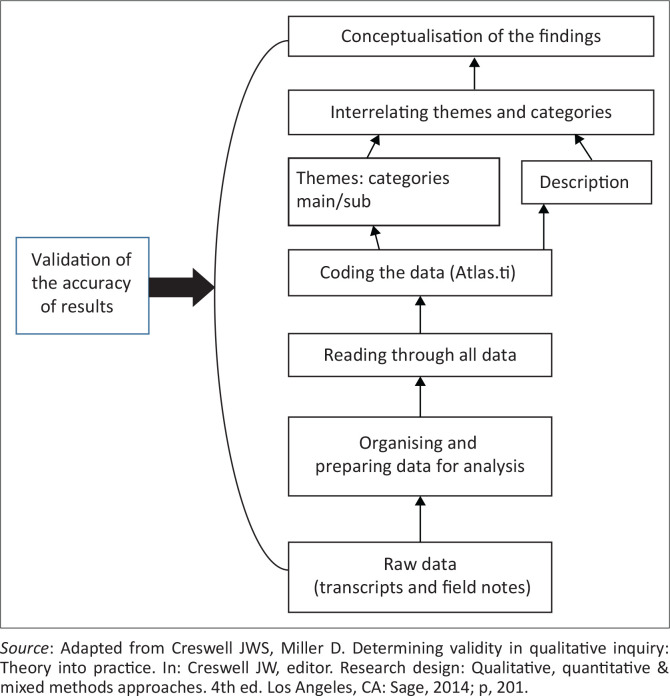
Data analysis process using the Creswell approach.

The audio recorded focus groups were transcribed verbatim. An independent coder experienced in qualitative data analysis was used to ensure trustworthiness. The collected data were organised and displayed in a fashion that provided answers to the research questions.^15^ This practice necessitated categorising, ordering, manipulating and summarising data in meaningful terms.^14,16,17^ Inductive and intuitive analysis, synthesis and derivation strategies were used by the researcher in the analysis of the collected data.^18^ In this process, the researcher acquainted herself with the collected data by repeatedly listening to the audio tapes and reading the transcripts, with the field notes obtained during the focus group interviews. A linear, hierarchical approach, building from the bottom to the top was used by the researcher in analysing the data collected from the focus group interviews.^14^ This approach follows seven steps from the bottom to the top, with an interactive process involving various interrelated steps, as shown in Figure 1.

### Trustworthiness and rigour

Codes for themes (divided into themes and categories) were used by the researcher in analysing the data and memos about the context and variations in the phenomenon under study. The researcher further verified the selected themes by reflecting on the data and through discussion with an independent coder, in order to refine categories and identify propositions. The services of an independent coder were engaged based on their experience and knowledge in qualitative research. Subsequent consensus discussions were held between the researcher and independent coder to determine and agree on similar patterns of themes that emerged from the focus group interviews. A follow-up interview with one group of participants was held to verify the findings and provide an opportunity to check the interpretation of the responses from the three interviews related to perceptions of the school health personnel on vision screenings in schools.^14^

Transferability was achieved through the dense description of demographic information and rich description of the data supported by the direct quotations of participants and results. The dense description of the research methodology ensures dependability and confirmability was ensured by using direct quotes from the participants.

### Ethical considerations

Ethical approval to conduct the study was obtained from the Research Ethics Commitee of the University of Johannesburg (ethical clearance number: AEC 01-09-2014). Gatekeeper approval, including that from the Gauteng Department of Health Protocol Review Committee, was obtained before commencing with the data collection (protocol number: P141013). The participants signed the informed consent to participate voluntarily with no incentive offered. Participants were informed of their right to withdraw from this study at any time should they wish to do so. Confidentiality in a focus group cannot be ensured; however, a request was made to the participants to adhere to confidentiality within the focus groups. Anonymity was maintained throughout the study process by allocating the code names to the participants.

## Results

Coding and analysis of the textual data were carried out using Atlas.ti, a computer software programme that facilitates the creation and assignment of codes to text. The coding and analysis processes were thematic, wherein recurring themes and patterns in the data were identified, categorised and explained. The main and subcategories were decided upon through a consensus discussion between the researcher and the independent coder, as shown in Table 1.^19^ The results are presented according to these categories.

**TABLE 1:** Themes and categories that emerged from focus group interviews.

Themes	Categories
Vision screeners training	Type of training received Adequacy of training Suggestions on training
Vision screening tests	Type of tests performed Vision screening criteria for learners Adequacy of the vision screening methods used Opinions on the Snellen chart Views on modern technology
Referrals	Referrals and follow-ups
Role players and coordination of school vision screening	Learners Schools/teachers Parents School health personnel Optometrists
Challenges in the administration of school vision screening	Human resource Time Space Budgetary Equipment Communication

Source: Adapted from Metsing TI. Strategies to improve school vision screenings at primary health care level in Gauteng (South Africa) [document on the Internet]. University of Johannesburg. 2018. [cited n.d.]. Available from: https://www.proquest.com/docview/2528177376?pq-origsite=gscholar&fromopenview=true

### Demographics of participants

The participants included 12 females and 1 male school health personnel with a mean age of 31 ± 3 years old. All participants were black people with ± 6 years of experience. The school health personnel referred to as SHNs who participated in the focus group discussions included 10 professional nurses, one school health promoter, one auxillary nurse and one school health co-ordinator.

### Theme 1: Vision screeners training

The participants shared information related to the training they received on conducting vision screening. An all-round response confirmed that vision-screening training was provided to participants by the Department of Health (DoH). Consistency in statements made by the participants related to the form of training received by the three groups of SHNs and health promoters, as follows: (1) coaching sessions by the district optometrists, (2) mentoring by supervisors and (3) peer coaching. As stated:

‘[*Y*]es, we actually received training once a year, where we were informed by optometrists about the conditions that affect the eyes and how to test vision. There is no special training we underwent, we have had a few hours of information sessions with an optometrist, who showed us how to measure the VAs [*visual acuities*].’ (SHN A, Ekurhuleni South District, 12 June 2017)

‘[*W*]e did not receive formal training, the training received was just basically to let us understand school health from the policy point of view and what was expected from us as school health nurses.’ (SHN M & I, Ekurhuleni North District, 12 and 19 June 2017)

‘[*Y*]es, we actually have received training maybe once a year. Where we were informed about the conditions affecting the eyes and how to test vision. Actually, the training is usually arranged for the newly employed school health nurses.’ (SNH A, Ekurhuleni South District, 12 June 2017)

‘[*W*]e learned how to do the tests to evaluate eye movements for identification of squints from our previous supervisor.’ (SHN N, Ekurhuleni South District, 12 June 2017)

### Theme 2: Vision screening tests

Even though the participants received informal training in the school vision screenings in the form of coaching, mentoring and peer coaching, they expressed the need for further training as follows:

‘[*M*]ore training from the ophthalmic point of view will make our screening better. We are seeing some pathological conditions for the first time, with training we can identify these conditions and refer the child accordingly. We sometimes don’t know how to describe some of them.’ (SHN A, Ekurhuleni South, 12 June 2017)

‘[*T*]raining is needed in order to know when to refer, we must know when to send the children identified to have problems to the hospital or to the clinic. Like, which cases are urgent or not?’ (SHN RD, Ekurhuleni North District, 19 June 2017)

‘[*W*]e only know that if the VA [*visual acuity*] measured is above 6/7.5 we have to refer the child to an optometrist, we don’t know the significance of the numbers.’ (SHN MC, Ekurhuleni North, 12 June 2017)

‘[*N*]o, we don’t know how to measure the nearpoint of convergence [*NPC*] or anything about that test.’ (SHN S, Ekurhuleni North, 12 June 2017)

‘[*W*]hen the space allocated by the school is small, the chart is just moved to 3 m and that makes it easier for us to measure the VAs.’ (SHN J, Ekurhuleni North, 12 June 2017)

Requests for further training on how to conduct the vision screenings were related to the following areas of knowledge:

- Conversion of Snellen chart VA in metre notation for a 3 m testing distance.
- Knowledge on the significance of the numbers (6/6, 6/7.5, …, 6/60) and thus interpretation when the VAs are evaluated.
- Accurate identification and description of ocular pathologies including identification of those needing urgent attention.
- The ability to do other tests when children complain of poor vision despite the VA measurements revealing a 6/6 vision such as the ability to measure at least one of the visual efficiency skills (e.g. nearpoint of convergence).

### Theme 3: Referrals and follow-ups

Another key discussion point regarding the school vision screening programme was on the referral criteria, mechanisms and procedures. Learners identified as having visual anomalies during the screening tests were said to be referred to ophthalmologists at the local district clinics. The point was also made that deciding on whether to refer a child or not was complicated by some uncertainty on the part of the nurses. Illustrated in the quotes:

‘[*A*]nything suspicious is referred to them … we sometimes don’t know if the children went to see the ophthalmologist or not, but that depends on the parents. Sometimes we are pushed to refer them to Ophthalmologists or Optometrists because they are complaining, even if we are not sure that they have a visual problem.’ (PN M, Ekurhuleni North, 19 June 2017)

‘[*W*]e refer children suspected to have visual problems, but we most of the time don’t get good feedback from the ophthalmologists or optometrists.’ (PN N, Ekurhuleni North, 19 June 2017)

Apart from insufficient feedback, another challenge relating to referrals was said to be that the children took long to be seen by the optometrists and to get spectacles once referred. In light of the various limitations inherent in the existing referral system, it is not surprising that a suggestion was made that the SHNs be equipped with more training and skills to remedy the identified problems themselves.

### Theme 4: Role players and coordination of school vision screening

Communication from learners to their parents, support from the schools was lacking, and the availability of optometrists was found to be lacking. Therefore, the following concerns were raised by the SHNs:

‘[*S*]ometimes school children do not give the referral letters or consent forms to their parents. These letters usually are either left in the bags or thrown in the streets.’ (SHN A, Ekurhuleni North, 12 June 2017)

‘[*T*]he problem we have with the consent forms is that some parents refuse to have their children’s vision screened, even though they have an obvious vision problem.’ (SHN J, Ekurhuleni North, 21 June 2017)

‘[*C*]ommunication is a problem we encounter when we visit the schools … we get no support from the teachers or the schools.’ (SHN R, Ekurhuleni South, 19 June 2017)

‘[*R*]eferrals to optometrists are a problem. It’s not happening in the way it should be happening. In the past we used to refer to the private optometrists such as SpecSavers etc. … But because of logistics we have been stopped and told not to refer to them because of the budget constraints and all. We therefore have been told to refer to our own district optometrists. But it’s still a challenge because of the long queues and sometimes facilities are very far for the children to reach.’ (SHN MQ, Ekurhuleni South, 12 June 2017)

As a result of the legal and ethical purposes, the vision screening could not be performed on any child without the written consent of their parents or legal guardians. Apart from insufficient feedback, another challenge relating to referrals was said to be that the children took long to be seen by the optometrists and to get spectacles once referred. In light of the various limitations inherent in the existing referral system, it is not surprising that a suggestion was made that the SHNs should be equipped with more training and skills to remedy the identified problems themselves.

### Theme 5: Challenges in the administration of school vision screening

In the process of discussing their vision screening functions, the participants expressed and highlighted a number of challenges they faced as follows:

‘[*O*]ur team is allocated a lot of schools, and we only have two teams split from a group of five nurses. We definitely need more school health nurses.’ (SHN Z, Ekurhuleni South, 12 June 2017)

‘[*V*]ision is not the only thing we screen … We have the weight, height, vision, oral hygiene etc … to screen.’ (SHN S, Ekurhuleni North, 21 June 2017)

‘[*I*] am also thinking about the other challenges we have in terms of space. You sometimes find that the rooms we have to conduct the health screenings in are very small and it becomes difficult to measure the 6 m.’ (SHN J, Ekurhuleni North, 21 June 2017)

‘[*W*]e have a lot of equipment and instruments to carry, lack of proper transportation hinders our performance.’ (SHN S, Ekurhuleni North, 21 June 2017)

‘[*S*]ome schools don’t even want to know how we work, but in other schools the teachers will come with the learner when they have the health screening.’ (SHN RD, Ekurhuleni North, 19 June 2017)

The given challenges ranged from human resources, material and financial constraints, including the coordination and communication problems in conducting effective vision screenings. There was a perception of insufficient nurse to learner ratio, thus creating heavy workloads for the currently employed SHNs. The shortage of SHNs was seen as not only putting a lot of work pressure on the nurses but also as potentially compromising the quality of the work being performed. Further compounding time pressures were space limitations that hampered the more efficient administration of the vision screening tests. Although not cited as frequently as other constraints, there was also some mention of budgetary limitations somewhat hampering the programme. Whilst communication was an issue pertaining to referrals and follow-ups with eye care professionals, challenges were further experienced in communicating with the school teachers assigned to co-ordinate the school health screenings and with the parents of children needing to be vision screened. Compounding the problem were issues relating to the comprehension of the messages sent to the parents, wherein participants cited the need for other languages to be used when the consent forms are drafted.

## Discussion

Impaired vision can affect a child’s neurological, emotional, cognitive and physical development by limiting the range of experiences and kinds of information that the child is exposed to.^20^ School health nurses are the core of school health programmes but can only fulfil this pivotal role with appropriate and adequate training. Vision screening training of school health personnel depends on national/state requirements, availability of professionals and volunteers, staffing patterns of school health programmes, equipment available and training.^21^ Inadequately trained school vision screening personnel may lead to high false-negative and false-positive results, which are significant drawbacks to vision screenings. However, capacitating the frontline role players to execute their due responsibilities may contribute positively to the fundamental success of the school health services, including vision screenings.

Participants reported being trained informally in conducting vision screenings. Following this informal training, the main concern in this area appears to relate to comprehensive training in the assessment and interpretation of VA measured with the Snellen chart. In addition, training in the ability to identify sight-threatening ocular pathology particularly those requiring urgent referrals, and other basic tests, for example, convergence tests that could affect learning in children, were expressed as important to the SHNs. The current vision screening protocol was the Snellen chart with alphabets and illiterate ‘E’ chart for distance VA measurement and direct observations of the eyes to detect ocular pathologies. Whilst distance VA can reliably detect myopia, it may miss hyperopia or astigmatism, important sources of visual discomfort in children of school-going age important in the delivering positive outcomes.^22,23^

Robinson and colleagues,^8^ in their study, concluded that public health nurses when trained to conduct vision screenings can effectively administer tests of VA, stereoacuity and ocular alignment.^24^ In South Africa, this type of training can be adequately provided by optometrists, particularly those with a focus on paediatrics. Thus, a recommendation on a wider scale for governments to develop minimum qualifications and provide ongoing, high-quality training involving various eye care sectors for vision screeners.^24^ Furthermore, the establishment of more consistent standards for training vision screeners and provision of support for continuing education in this field should be considered.

Poor communication in schools emerged from the focus group interviews. These statements indicate that there is a need for cooperation amongst all the role players, namely the SHNs, schools/teachers, parents and learners. According to the ISHP implemented in 2012, the co-ordination of school health is performed by the DoH.^4^ The DoH is responsible for the provision of school health services, and the DoBE (Department of Basic Education) plays a key role in creating an enabling environment for the provision of the ISHP. This includes planning, managing and monitoring of the programme, facilitating access to schools and services and liaising with other role players at all levels of the system. Thus, a structured collaboration between these departments is necessary, even though the implementation of the ISHP at the school level is the responsibility of the School-Based Support Team (SBST) under the guidance of the school principal. The school SBSTs include life skills/orientation teachers, members of the school health team (including health promoters), representatives from the school governing body, representatives of relevant non-governmental organisations (NGOs) and community-based organisations (CBOs), peers and learners.^4^

Notably, challenges cited by participants included an absence of cooperation from parents to sign consent forms, space limitations and lack of feedback from eye care professionals. There is a need for improved cohesion and communication between all role players including parents to enable reasonable and professional provision of validated vision screening services that have the best chance of detecting children with visual anomalies. Effective vision screening is characterised by follow-up care for those children who have failed the screening test and may need corrective lenses or therapy. The delay in initiating treatment of visual problems after a referral letter is given to the learner was cited by SHNs as being because of several factors, including children not giving the referral letters to their parents, parents not acting and sending their children for further professional care and district optometrists being fully booked at the clinics.

Human resource constraints were reported by the participants with respect to the need to increase the number of school health teams per district because of insufficient nurse to learner ratio, thus creating heavy workloads. The addition of another test was viewed as a challenge of coverage requiring an increased number of teams and accompanying cost implications. According to the ISHP in each municipality or district, 2000 learners per year are expected to be visually screened by each nurse.^2^ In a country such as India, which experiences similar concerns of shortages of vision screening personnel, school teachers are trained to deliver school children’s eye screenings (SCEs).^25^ The SCES programme was found to be very effective in terms of coverage and less costly compared with the primary healthcare (PHC) model. In addition, the involvement of teachers in vision screenings was found to have improved compliance with referrals and follow-ups for children detected to have visual problems.^4,26^ Other studies argued that with the high teaching workloads and administrative tasks in schools, it might not be feasible to engage the teachers in vision screenings.^27^ The staffing patterns of school health programmes depend on a needs-analysis, availability of the human resources and financial resources. Evaluation of the personnel involved in school vision-screenings needs to be reviewed.

Lack of uniformity emerged from focus group interviews regarding the criteria for referral. Variable opinions exist on the most suitable VA level regarded as the cut-off for referral because of lack of data on the impacts of mild vision impairment on functional and quality of life (Centre for Community Child Health, 2008).^28^ In most countries, the criteria for failure of the vision screening is VAs worse than 20/40 (6/12) for the pre-schoolers, worse than 20/30 (6/9) for those ≥ 6 years including a two-line or more difference between the two eyes are considered (Colorado, Iowa, Alaska, Pennsylvania and New York).^29,30,31,32,33^ However, in California, the criteria for failure of vision screening is VAs worse than 6/15 (20/50) for children ≤ 6 years and for older children ≥ 6 years, VAs worse than 6/9, including the two-line difference between the two eyes. In the United Kingdom, the criteria for failure of vision screening is VAs of 6/24 or worse and the interocular difference of more than 0.075 (i.e. a two-line difference in VAs between the two eyes).^34^

The appropriate referral (or pass/fail) criteria for use in vision screening in most countries is dependent on the age of the child screened.^35^ A change of the screening criteria from 6/7.5 (LogMar 0.1) to 6/9.5 (LogMar 0.2) was found to substantially increase the number of over-referrals and lead to the prescription of unnecessary spectacles, leading to failure to identify children who might benefit from glasses. In other studies, it was concluded that the lower the criteria (e.g. worse than 6/7.75 or 6/6), the higher the false referrals and the higher the criteria (e.g. or 6/9.5 or 6/12), the lower the false referrals. Therefore, selection of proper referral criteria is essential despite the worldwide differences in criteria used to determine fail or pass in vision screenings.^36,37,38^

Regardless of the lack of data on the impacts of mild vision impairment on functional and quality of life, the referral criteria used in school health policies should consider the avoidance of false referrals. As these can be costly to the government and consider the referral criteria of VAs worse than 6/12 for the pre-schoolers and VAs worse than 6/9 for the older children or two lines of difference between the eyes.^36^ There is a need for the criteria for referral to be standardised.

Participants cited referrals and follow-ups as challenges. Participants reported problems relating to referral letters not given to parents by learners, feedback not given back to them by the district optometrists and limited availability of convenient eye care appointments.^4^ Follow-up care for children who have failed the screening test is an important part of the vision screening programme, enabling those with visual anomalies to receive corrective lenses or therapy.^28,39^ Non-compliance with follow-ups after visual screening failure is a recognised impediment to the care of untreated vision anomalies and is often found to contribute towards children struggling with their academic performance.^20^

Other barriers included unavailability of the care givers or parents to take children identified with visual anomalies for follow-up care because of work and family issues, such as a large family or disabled parents.^40^ Perceptual barriers exist related to parents not believing that their children had visual problems and thus not prioritising eye examinations. Other studies reported that the greatest barrier to follow-up care was because of the inability to contact families.^38,40^ Intervention strategies were developed to remedy the situation through making immediate arrangements for follow-up care, on-site visual assessment, increased follow-ups by the programme director and lastly offering logistic support to families such as providing transport to eye appointments.^41,42^

### Limitations of the study

The SHNs were the only participants involved in the collection of qualitative data and teachers and parents were not invited. The perceptions and attitudes of the teachers and parents could have provided the researcher with information related to their attitudes, perceptions and suggestions on how to improve the school vision-screening programme.

### Recommendations

The study also did not attempt to determine the feasibility of involving other personnel (such as lay persons and teachers) in conducting the school vision screenings besides the SHNs, in view of the fact that they are few and overworked.

## Conclusion

The involvement of eye care professionals in the development of the vision screening guidelines, including determination of the criteria for referral, age at which the vision screenings are to be conducted and the follow-up activities, can address the challenges currently experienced with the implemented Integrated School Health Policy (2012). Follow-up practice of children detected to have visual anomalies is important for an effective vision screening. Thus, improved cohesion and communication between all role players will enable the reasonable and professional provision of validated vision screening services that have the best chance of detecting children with vision anomalies.
